# On the Importance of Halogen and Chalcogen Bonds in the Solid State of Nucleic Acids: A Combined Crystallographic and Theoretical Perspective

**DOI:** 10.3390/ijms241713035

**Published:** 2023-08-22

**Authors:** María de las Nieves Piña, Antonio Bauzá

**Affiliations:** Departament de Química, Universitat de les Illes Balears, Ctra. de Valldemossa Km 7.5, 07122 Palma de Mallorca, Baleares, Spain; neus.pinya@uib.es

**Keywords:** nucleic acids, PDB survey, solid state chemistry, halogen bonding, chalcogen bonding, theoretical study

## Abstract

In this work, intra- and intermolecular halogen and chalcogen bonds (HlgBs and ChBs, respectively) present in the solid state of nucleic acids (NAs) have been studied at the RI-MP2/def2-TZVP level of theory. To achieve this, a Protein Data Bank (PDB) survey was carried out, revealing a series of structures in which Br/I or S/Se/Te atoms belonging to nucleobases or pentose rings were involved in noncovalent interactions (NCIs) with electron-rich species. The energetics and directionality of these NCIs were rationalized through a computational study, which included the use of Molecular Electrostatic Potential (MEP) surfaces, the Quantum Theory of Atoms in Molecules (QTAIM), and Non Covalent Interaction plot (NCIplot) and Natural Bonding Orbital (NBO) techniques.

## 1. Introduction

Noncovalent forces are of pivotal significance in biology and compose the fundamentals of modern chemistry [1,2,3,4]. This has led to an increase in the number noncovalent interactions (NCIs) characterized to date, which exert a crucial role in several biological mechanisms, such as enzyme inhibition [5], ion transport [6], and protein folding [7]. In a parallel way to the well-known hydrogen bonds (HBs) [8], halogen bonds (HlgBs) are nowadays a well-established and well-studied NCI among supramolecular chemists [9,10,11,12,13,14,15]. Both binding forces have been recently described as members of the ‘σ-hole family’ [16,17,18]. A σ-hole was originally conceived as a region of positive electrostatic potential located on the extension of a covalent bond. For instance, σ-holes can be found along the Se–F/C–Br bonds in difluoro selenide or bromoethane molecules. In the case of halogens, the σ-hole donor ability increases from F to I, resulting in a strengthening of the interaction upon descending in the group.

In biological systems, halogen atoms are typically used in the field of drug design and to probe molecular interactions [19,20], while, on the other hand, halogenated bases are useful molecular synthons for solving the X-ray crystal structures of nucleic acids (NAs) using the Multiwavelength Anomalous Dispersion (MAD) technique [21]. In this regard, several examples exist in the literature where HlgBs are functional and relevant binding forces, such as works by Carter [22] and Voth [23] and collaborators in which HlgBs were engineered for studying DNA Holliday junctions. In addition, the theoretical reports from Parker [24] and Xu [25] and collaborators unraveled the potential role of HlgBs as a relevant interaction in both NA structures and base pair stabilization.

Furthermore, Enninfar and coworkers [26] utilized a variety of experimental techniques (e.g., steady-state fluorescence, UV thermal melting, X-ray crystallography, and gel electrophoresis) to understand the influence of halogenation (by incorporating either a bromine or iodine) on RNA folding. They demonstrated that the RNA hairpin/duplex ratio was drastically influenced by both the presence and position of halogenation along the RNA chain. Moreover, Kolář [27] and Auffinger [28] and collaborators have reported statistical evidence of the promising potential of HlgBs involving NAs. Our group has also contributed to this field with three computational studies, involving intermolecular protein-NA HlgBs [29], quantification of intramolecular halogenated nucleotide···phosphate HlgBs [30], as well as evaluating the effect of NA halogenation on the stability of canonical and noncanonical base pairs [31].

On the other hand, the use of chalcogen compounds in biology (mainly Se and Te) has been related to the field of rational drug design and, more specifically, to the development of noncancer therapeutic agents [32]. Despite this, studies have also demonstrated the implications of chalcogen bonding interactions (ChBs) [33,34] in several biological phenomena, such as the stabilization of protein structures [35,36,37] or the formation of RNA–ligand and protein–ligand complexes [38,39,40]. In addition, Se and Te have been also incorporated into the structures of NAs for phase and structure determination in nucleic acid X-ray crystallography [41,42,43], similar to the replacement of methionines by selenomethionines in proteins. In this context, a recent study carried out by Sharma and collaborators [44] proposed the use of ChBs as an additional source of stabilization in NA base pairs.

**Figure 1:**
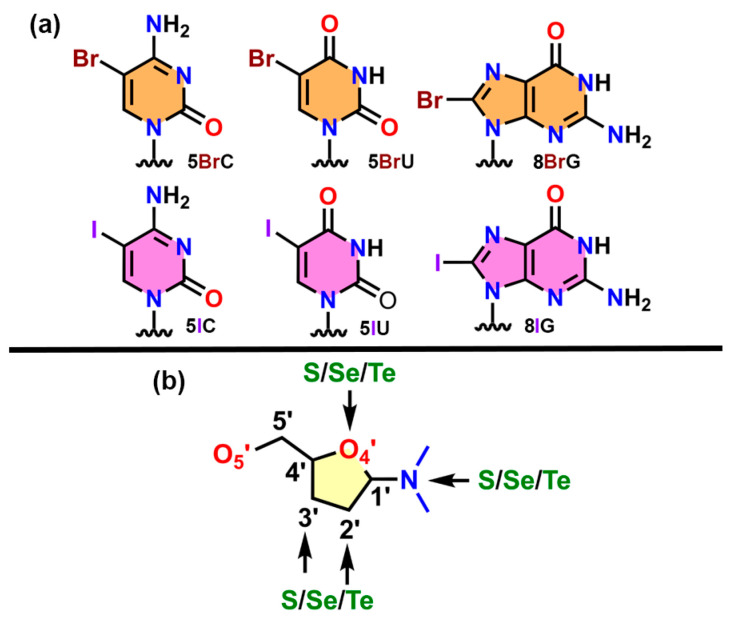
(**a**) Schematic representation of the halogenated nucleobases considered in the PDB survey (5BrC, 5IC, 5BrU, 5BrI, 8BrG, and 8IG represent 5-bromocytosine, 5-iodocytosine, 5-bromouracil, 5-iodouracil, 8-bromoguanine, and 8-iodoguanine, respectively); (**b**) Schematic representation of the nucleotide positions considered for the incorporation of S, Se, or Te atoms.

Building upon these previous reports, our aim was to investigate the plausible structural role of HlgBs and ChBs in the solid-state architecture of isolated NAs. To reach this goal, we conducted a search in the Protein Data Bank (PDB) [45], retrieving a total of 11 nucleic acid structures exhibiting highly directional HlgBs and ChBs in their solid-state architecture (see Figure 1 for the halogenated and chalcogenated molecules used). These contacts corresponded to either an intramolecular (e.g., within the same NA strand) or intermolecular (involving two neighboring NA chains) interaction and assisted in crystal packing formation. Calculations at the RI-MP2/def2-TZVP level of theory were conducted to shed light into the strength, directionality, and physical nature of the HlgBs and ChBs present in these structures by means of the Molecular Electrostatic Potential (MEP), the Quantum Theory of Atoms in Molecules (QTAIM), and NCIplot (Non Covalent Interaction plot) and Natural Bonding Orbital (NBO) techniques. We believe that the results derived from this study might be important to those scientists working in the fields of NA or protein engineering and σ-hole-based chemistry, as well as in the development of NA-based materials.

## 2. Results and Discussion

### 2.1. Results from the PDB Survey: Br and I Halogen Bonds

**Figure 2:**
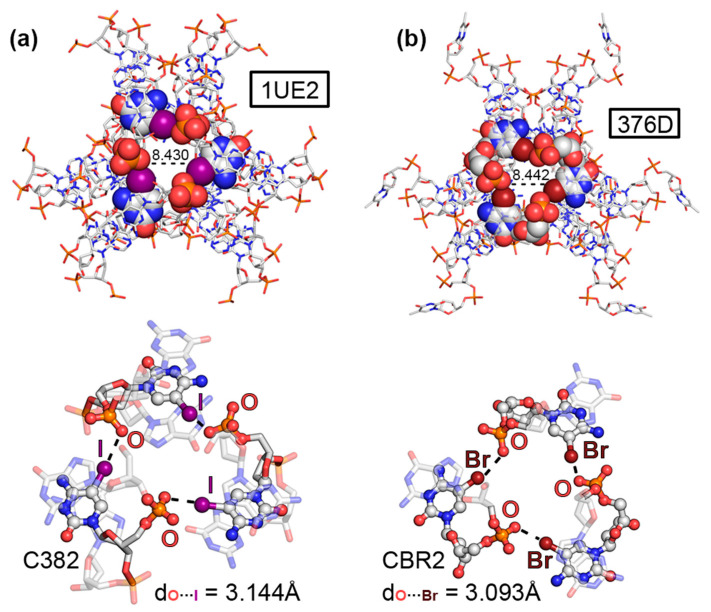
Partial views of the X-ray structures of (**a**) 1UE2 and (**b**) 376D with the molecular cavity width indicated (in Å). The HlgBs are magnified in the bottom part of the figure with the O···I and O···Br distances indicated.

Four examples were retrieved from the PDB inspection involving halogenated nucleobases. The first two corresponded to structures 1UE2 [46] and 376D [47], involving two DNA fragments containing the sequences d(G^I^CGAAAGCT) and d(G^Br^CGAAAGCT), respectively. Studies have demonstrated that similar sequences exhibit unconventional properties such as (i) abnormal mobility in electrophoresis [48], (ii) high thermostability [49], (iii) uncommon circular dichroism spectra [49], as well as (iv) unusual resistance against nuclease digestion [50]. In both cases, 5IC and 5BrC were used during the X-ray diffraction process by means of the MAD method [21]. A close inspection of both solid-state architectures revealed the formation of DNA helical duplexes in parallel disposition to the *z*-axis of the crystal lattice. This style of packing has been well studied in A and B NAs [51,52], and it is considered an important stabilizing effect in the crystal formation between two aligned symmetry-related helices. The DNA duplexes were associated in the form of a trimer, forming a molecular cavity (around 8.5 Å) in the middle of the structure (see Figure 2a,b). Interestingly, three symmetrically distributed HlgBs were established at the top and bottom parts of the assembly. These involved either iodine (in 1UE2) or bromine (in 376D) atoms acting as σ-hole donors and O atoms from vicinal phosphate groups as σ-hole acceptors, exhibiting O···I and O···Br distances shorter than the sum of the O/I and O/Br van der Waals radii (3.144 Å and 3.093 Å, respectively) and high directionality (O···I–C and O···Br–C angles of 168.3° and 162.2°, respectively). Theoretical calculations at the RI-MP2/def2-TZVP level of theory on a dimeric model revealed individual HlgB strengths of −16.6 and −12.8 kcal/mol, respectively, in line with the results obtained for charge-assisted HlgBs [53]. The presence of these three centered HlgBs was important for holding the DNA duplexes together, thus being a nice example of the potential application of HlgBs as a stabilizing agent in NA crystal structures.

**Figure 3:**
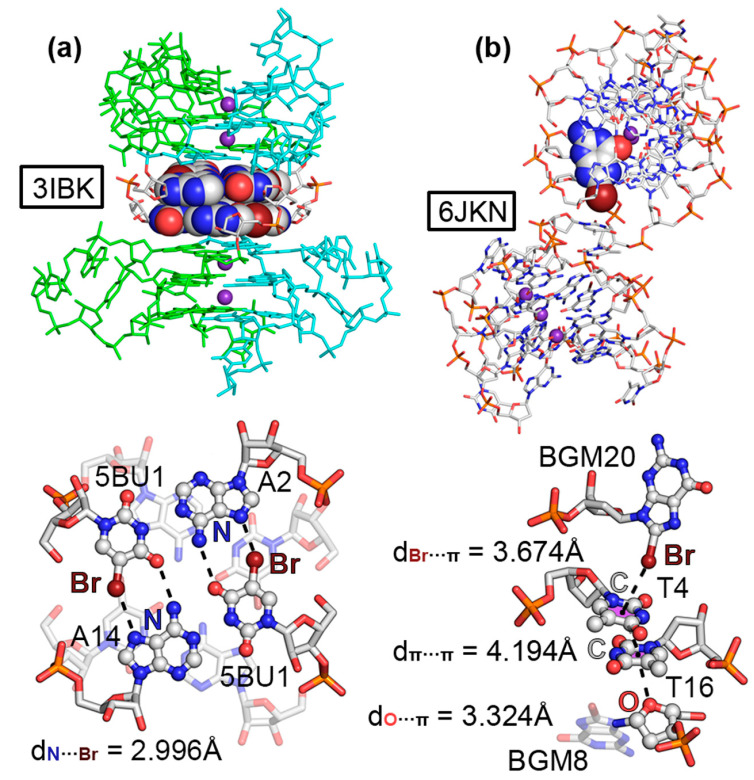
Partial views of the X-ray structures of (**a**) 3IBK and (**b**) 6JKN. In 3IBK, the two RNA chains are colored green and ice blue for easier identification. K^+^ ions are colored as purple spheres. The HlgBs are magnified in the bottom part of the figure with the N···Br and π···Br distances indicated.

The third example encompassed structure 3IBK, which corresponded to a telomeric RNA G-quadruplex with the sequence (U_Br_AGGGUUAGGGU) [54]. G-quadruplexes are part of the noncanonical NA family of structures and are formed by the stacking of G-quartets, which are planar arrangements of four guanines held together by several HBs [55,56]. In the crystal packing of 3IBK, the asymmetric unit was composed by two RNA strands (colored green and ice blue in Figure 3a, respectively), each of which contained two G-rich repeats, folding into a parallel four-stranded bimolecular G-quadruplex. The structure also contained K^+^ ions in the center of the channel, positioned between the three G-quartets. Interestingly, 5BrU was incorporated into the RNA sequence, being crucial for the enhancement of crystal diffraction quality by holding the dimeric interface tightly together, as stated by the original authors. A close inspection of the structure revealed the formation of a mixed HB/HlgB base pair, involving the Br and O atoms belonging to 5BrU and the N_7_ and −NH_2_ group from adenine, located at the interface between two G-quadruplexes (see Figure 3a bottom for a detailed view of the interaction). The computed interaction energy of this noncanonical base pair reported a strength of −6.0 kcal/mol (in line with our recent study [31]) and a HlgB energetic contribution of −2.2 kcal/mol. Owing to the short N···Br distance (2.996 Å) and the directionality observed (157.2°), this example illustrates the potential role of halogenated base pairs in the stabilization of noncanonical nucleic acid structures.

The last selected structure corresponded to a bromo G-quadruplex of human telomeric DNA (6JKN) [57]. In this example, the crystal packing was composed of discrete G-quadruplex molecules, with K^+^ ions inserted in the center of each unit in a similar fashion to that observed in the 3IBK structure. These isolated units interacted with each other to form a supramolecular assembly. In Figure 3b, a G-quadruplex dimer is highlighted, in which the Br atom belonging to 8BrG from one unit (BGM20) interacted through a HlgB with the π-system of a thymine (T4) belonging to another G-quadruplex, exhibiting a C···Br distance of 3.674 Å. This type of HlgB has been also described in the literature as a halogen–π interaction [58]. In addition, a second thymine ring (T16) interacted with T4 though a π–π stacking interaction (π–π distance of 4.194 Å) and, lastly, a lone pair–π (lp–π) interaction was undertaken between the sp^3^ O from a sugar moiety belonging to a guanine base (BGM8) and the π-system of T16, with an intermolecular distance of 3.324 Å. Computations on a dimeric model consisting of BGM20 and T4 revealed a HlgB strength of −2.2 kcal/mol. The formation of this supramolecular assembly contributed to the stabilization of the G-quadruplexes in the solid state, serving as an example of the cooperation between different NCIs in the formation of solid-state NA structures.

**Figure 4:**
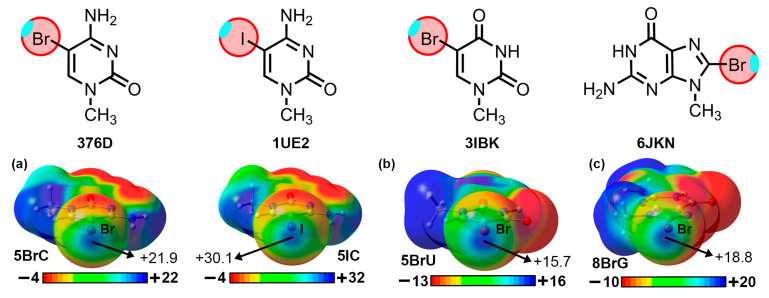
Molecular Electrostatic Potential (MEP) surfaces of (**a**) 5BrC and 5IC, (**b**) 5BrU, and (**c**) 8BrG. Energy values at concrete regions of the surface are given in kcal/mol (0.001 a.u.). The MEP minima and maxima have been adjusted for clear visualization of the Br and I σ-holes. The corresponding PDB codes are also included below the schematics of each molecule.

To demonstrate the implication of the Br and I σ-holes in the HlgBs described above, we have computed the Molecular Electrostatic Potential (MEP) surfaces of 5BrC, 5IC, 5BrU, and 8BrG at the RI-MP2/def2-TZVP level of theory (see Figure 4). As noted, in all cases, a positive potential surface was observed along the prolongation of either the C–Br or C–I covalent bonds (σ-hole), with values ranging between +15.7 and +30.1 kcal/mol. Also, in the case of 5BrC and 5IC, the iodinated derivative exhibited a more positive σ-hole potential, in line with the larger polarizability exhibited by the I atom, as it is commonly known [17]. The presence of these electrophilic regions made these biological synthons suitable for favorable interactions with electron-rich species (e.g., lone pair-bearing molecules or π-systems), such as the ones described in the structures shown above.

As a final remark, the BSSE-corrected interaction energies, distances, and angle values corresponding to the HlgBs present in the highlighted structures are gathered in Table 1.

### 2.2. Results from the PDB Survey: S, Se, and Te Chalcogen Bonds

**Figure 5:**
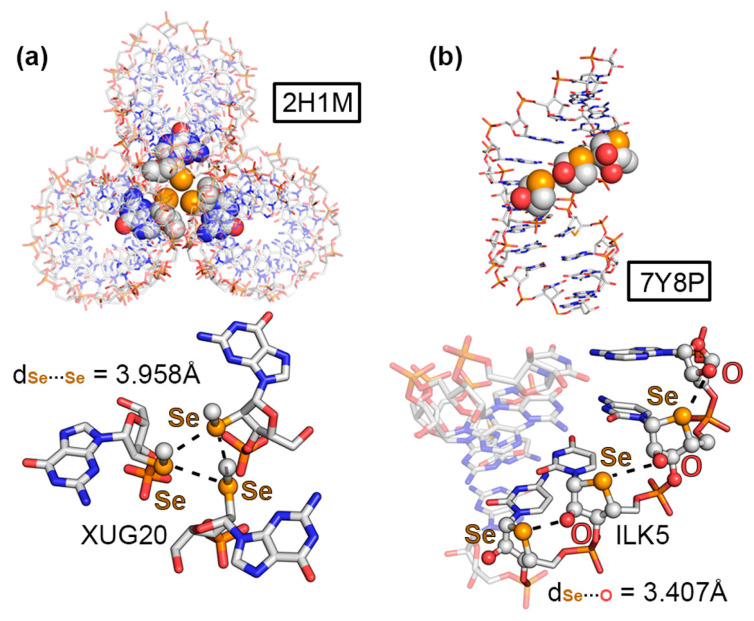
Partial views of the X-ray structures of (**a**) 2H1M and (**b**) 7Y8P. The ChBs are magnified in the bottom part of the figure with the Se···Se and O···Se distances indicated.

The first selected example corresponded to the 2H1M structure [59], which was composed of an RNA containing 2′-methylseleno guanosine. In more detail, the authors crystallized a 2′-methylseleno guanosine phosphoramidite by incorporating a methylseleno moiety in the 2′ position of the pentose ring. The solid-state architecture of this compound revealed the formation of a helical RNA duplex, which established noncovalent contacts with two other vicinal units (see Figure 5a). Interestingly, the central region of the RNA helical triad was mainly stabilized by three “like-like” ChBs between the 2′-methylseleno moieties, exhibiting equivalent Se···Se distances (3.958 Å). Theoretical calculations revealed a strength of −3.7 kcal/mol per ChB. This led to the formation of an equilateral triangle, which held the three RNA molecules together, thus significantly contributing to the observed packing structure.

The second structure (7Y8P, Figure 5b) corresponded to a 4′-seleno-modified RNA [60], which crystallized in the form of a duplex, maintaining the A-conformation observed in natural RNA with almost all of the ribonucleosides exhibiting north-type sugar puckering. Interestingly, the 4′-seleno derivatives were involved in three consecutive O···Se ChBs involving the 3′ OH group from a vicinal nucleotide within the same RNA strand; therefore, they acted as both σ-hole donor and acceptor counterparts. The computed interaction strength of one of these intramolecular ChBs (exhibiting a O···Se distance of 3.407 Å) resulted in −3.1 kcal/mol.

**Figure 6:**
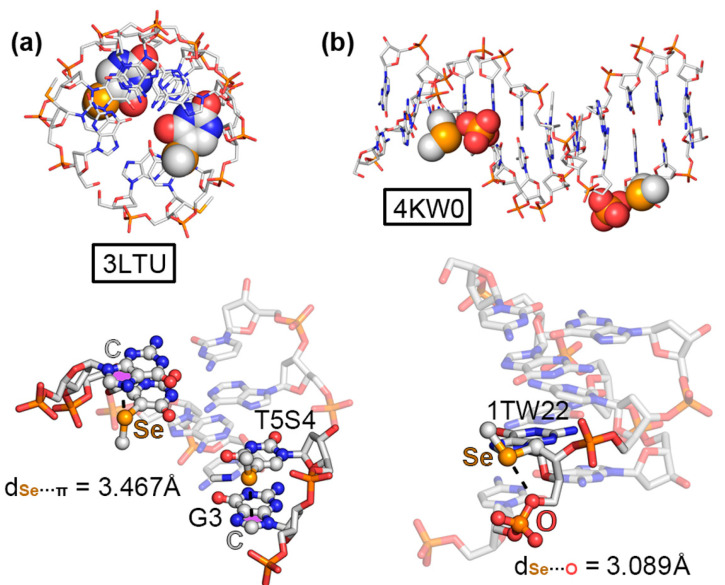
Partial views of the X-ray structures of (**a**) 3LTU and (**b**) 4KW0. The ChBs are magnified in the bottom part of the figure with the π···Se and O···Se distances indicated.

As the two last ChB examples, structures 3LTU [61] and 4KW0 [62], involved a DNA octamer containing 5-methylseleno-deoxyuridine and a DNA dodecamer containing 2′-methylseleno-guanine, respectively. As can be observed in Figure 6a,b, the methylseleno group established a ChB with (i) the π-system of a guanine ring (G3 in Figure 6a) and (ii) the lone pairs of a negatively charged O atom from a vicinal phosphate group (Figure 6b), with π···Se and O···Se distances of 3.467 and 3.089 Å, respectively. These interactions contributed to the stabilization of the DNA assemblies, with energetic strengths of −7.8 and −4.3 kcal/mol, serving as an alternative to classical HB and π–π stacking interactions.

In Table 2, the interaction energies, distances, and angle values with respect to the four discussed ChB examples are shown. In addition, we have also included additional NA structures retrieved from the PDB search that also exhibited directional ChBs in their solid-state packing (see ESI), such as 3DW6 [63], showing a N···Se ChB; 3HG8 [64] exhibiting a S···π ChB; and finally 3FA1 [65], which presented both Te···π and O···Te ChBs.

**Figure 7:**
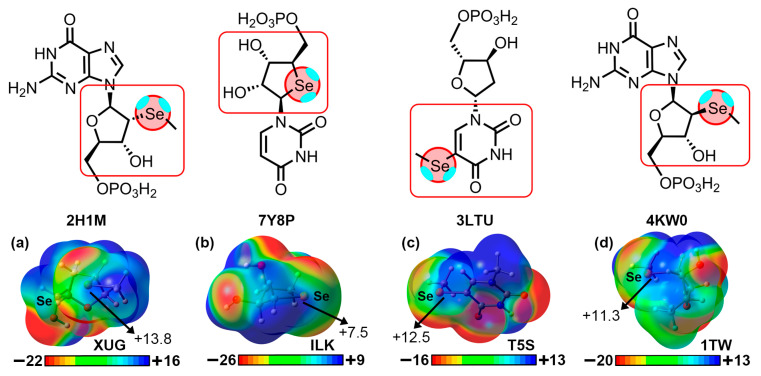
Molecular Electrostatic Potential (MEP) surfaces of the Se moieties present in (**a**) XUG, (**b**) ILK, (**c**) T5S, and (**d**) 1TW ligands. Energy values at concrete regions of the surface are given in kcal/mol (0.001 a.u.). The MEP minima and maxima have been adjusted for clear visualization of the Se σ-hole. The corresponding PDB codes are also included below the schematics of each molecule. The red squares indicate the portion of each ligand used for computing the MEP surface (see Figure S1 in ESI for the results involving the 3FA1, 3HG8, and 3DW6 structures).

We have also computed the electrostatic potential surfaces of the four selenated derivatives implied in the structures described in Figure 5 and Figure 6. As noted in Figure 7, in all cases, the Se atom exhibited an anisotropic distribution of the electron density, with two regions of high electron density corresponding to the two lone pairs and two regions of low electron density (σ-holes) located on the prolongation of the Se–C covalent bonds (only one is shown in Figure 7), as expected for this group [17]. The values of the electrostatic potential ranged from +7.5 to +13.8 kcal/mol, leading to the attractive energy values gathered in Table 2.

### 2.3. QTAIM and NCIplot Analyses

**Figure 8:**
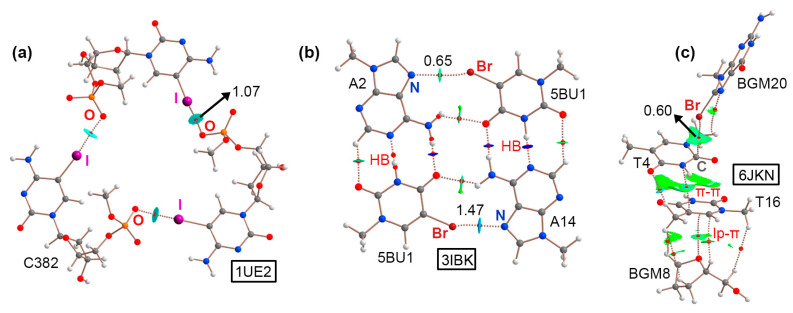
NCIplot analysis and AIM distribution of intermolecular bond critical points (BCPs, shown as red spheres) and bond paths in the (**a**) 1UE2, (**b**) 3IBK, and (**c**) 6JKN structures. Additional interactions present in these assemblies are denoted in red. The density values at the BCPs characterizing the HlgB interactions are also indicated. NCIplot surfaces involve only intermolecular contacts. NCIplot color range −0.02 au ≤ (signλ_2_)ρ ≤ +0.02 au. Isosurface value |RGD| = 0.5 and ρ cutoff = 0.04 au.

**Figure 9:**
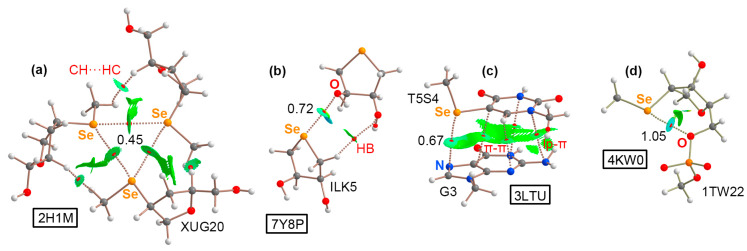
NCIplot analysis and AIM distribution of intermolecular bond critical points (BCPs, shown as red spheres) and bond paths in the (**a**) 2H1M, (**b**) 7Y8P, (**c**) 3LTU, and (**d**) 4KW0 structures. Additional interactions are denoted in red. The density values at the BCPs characterizing the ChB interactions are also indicated. NCIplot surfaces involving only intermolecular contacts. NCIplot color range −0.02 au ≤ (signλ_2_)ρ ≤ +0.02 au. Isosurface value |RGD| = 0.5 and ρ cutoff = 0.04 au.

With the purpose of characterizing the HlgBs and ChBs present in the PDB structures shown above from a charge density perspective, QTAIM [66] and NCIplot analyses were performed (see Figure 8 and Figure 9). As noted, in the case of the halogen-bonded complexes (Figure 8), the QTAIM analysis revealed a series of bond critical points (BCPs) and bond paths that characterized each assembly. In the 1UE2 structure (Figure 8a), three symmetrically distributed BCPs connected the iodine atom from 5IC to an O atom belonging to a phosphate group from a vicinal nucleic acid chain, leading to the formation of a supramolecular triangle directed by O···I HlgBs.

Structure 376D exhibited the same HlgB pattern, but in this case, the HlgBs involved 5BrC instead (see Figure S2 in ESI). In the case of the 3IBK structure (Figure 8b), six BCPs and bond paths characterized HBs from two “Watson–Crick” base pairs (disposed in a vertical fashion) between adenine and 5BrU. However, two BCPs and bond paths described a mixed HB/HlgB base pair that involved the “Hoogsteen” face of adenine (N_7_ and the –NH_2_ group) and the Br and O atoms from 5BrU (disposed in an horizontal arrangement), leading to the formation of a O···HN HB and a N···Br HlgB. The formation of this mixed HB/HlgB base pair interaction is in line with recent results reported by our group [31].

Lastly, in the case of the 6JKN structure (Figure 8c), the QTAIM analysis involved an assembly of NCIs spawning from an HlgB involving the π-system of thymine (T4) as an electron donor moiety (revealed by the BCP and bond path connecting the Br and C atoms from both counterparts), a π–π stacking interaction between two thymine rings (T4 and T6, mainly involving a C···N BCP), and finally, a lp–π interaction between T16 and the sp^3^ O atom from a pentose ring (denoted by the BCP and bond path connecting the O with a C atom from the nucleobase).

The ChBs present in structures 2H1M, 7Y8P, 3LTU, and 4KW0 were also analyzed from a charge density point of view (see Figure S2 in ESI for results involving the 3FA1, 3HG8, and 3DW6 structures). In the case of 2H1M (Figure 9a), three concomitant Se···Se ChBs were characterized by the presence of three BCPs and bond paths connecting the central Se atoms. In this structure, each Se atom acted as both Lewis acid (σ-hole donor) and Lewis base (σ-hole acceptor), being a unique example of how Se ChBs can modulate the solid-state architecture of nucleic acids. In addition, ancillary CH···HC interactions were also denoted by the presence of a BCP and bond path connecting the CH groups from the Se–CH_3_ moiety and the sugar ring.

In the case of the 7Y8P structure (Figure 9b), the analysis was carried out on a dimer, and a O···Se ChB was characterized by the presence of a BCP and bond path connecting both atoms. In addition, an ancillary HB was also denoted by the presence of a BCP that connected a CH group and an O atom from the two selenated sugar moieties. 

In 3LTU (Figure 9c), several BCPs and bond paths were observed, characterizing (i) a ChB involving the σ-hole present in the Se–CH_3_ moiety and the N atom from the five-membered ring belonging to guanine, (ii) a π–π stacking interaction denoted by the presence of three BCPs and bond paths that connected both nucleobases, and (iii) a lp–π bond that involved the sp^3^ N atom from guanine and the π-system of the selenated nucleobase.

Finally, in the 4KW0 structure (Figure 9d), an intramolecular ChB was denoted by the presence of a BCP and bond path connecting the σ-hole present in the Se–CH_3_ moiety attached to the pentose ring and an O atom from a neighboring phosphate group. QTAIM analyses of the 376D, 3DW6, 3HG8, and 3FA1 structures are included in Figure S2 (see ESI), exhibiting BCPs and bond paths that characterized (i) three symmetrical O···Br HlgBs (376D), (ii) a π···S/Te ChB (3HG8 and 3FA1), and (iii) a O···Te ChB (3FA1). In the case of 3DW6, no N···Se BCP was found and the interaction was analyzed by means of the NCIplot visual index.

In this regard, the NCIplot analysis revealed a greenish (in 6JKN, 2H1M, 7Y8P, 3LTU, 3DW6, and 3HG8) or bluish (in 1UE2, 376D, 4KW0, and 3FA1) isosurface between the halogen/chalcogen atoms and the electron-rich moiety, thus confirming the attractive and weak/moderate nature of the HlgB and ChB interactions studied herein along with their extension in real space (see Figure S2 in ESI for the NCIplot analyses of the 3FA1, 3HG8, and 3DW6 structures).

### 2.4. NBO Analysis

At this point, we were also curious about the orbital interactions responsible for the stabilization of the HlgB and ChB complexes studied herein. Thus, the NBO approach was used with particular emphasis on the second-order perturbation analysis [67] of the structures discussed above, owing to its usefulness when studying donor–acceptor interactions (see Table 3). As noted, in structures 376D, 3IBK, and 1UE2 (corresponding to HlgB complexes), the orbital contribution that characterized the interaction corresponded to the donation from either a N or O lone pair (LP) to an antibonding (BD*) Hlg–C orbital (Br–C/I–C). In the case of the 6JKN structure, the NBO analysis revealed a donation from the π-system of the thymine ring, involving a bonding (BD) C=O orbital to an antibonding (BD*) Br–C orbital.

On the other hand, in 2H1M, 7Y8P, and 4KW0, a contribution from a LP of the Se/O atoms to an antibonding (BD*) Se–C orbital was observed. In addition, in 3LTU, the donating orbitals involved the π-system of a selenated thymine (BD C–C and BD C–N), which interacted with antibonding (BD*) Se–C orbitals. The magnitude of these orbital interactions ranged between 3.29 and 0.14 kcal/mol, representing 10–60% (in the case of the HlgB complexes) and 5–25% (in the case of ChB complexes) of the total interaction energies gathered in Table 1, thus playing a moderate role in the stabilization of the HlgBs and ChBs studied herein. The differences observed in the magnitude of the orbital contribution for both interactions might be attributed to (i) the shorter distances exhibited by the HlgB complexes (all of them below 3 Å except for the 6JKN structure) as well as (ii) their higher directionality (between 157° and 175°) compared to the ChBs (between 148° and 173°).

## 3. Materials and Methods

### 3.1. Protein Data Bank Search Criteria

The PDB was inspected in June 2023 to find nucleic acid structures in which HlgBs and ChBs were established in their solid-state architecture. To achieve this, the following criteria were used:

- Only isolated nucleic acid X-ray structures were considered;
- Structures with disorder were not considered;
- In the case of halogens, only the incorporation of Br/I in the nucleobase (U, C, and G) was taken into account, while for chalcogens, the incorporation of S/Se/Te was considered in both the nucleobase and the sugar moiety (see Figure 1 above);
- Any type of nucleic acid structure (both canonical and noncanonical) was considered.

The application of these criteria yielded a total of 134 (Br and I), 22 (S), 54 (Se), and 3 (Te) X-ray structures. These were manually inspected for the presence of highly directional HlgBs and ChBs using the following geometrical criteria:

Distance criteria: d_A···X_ ≤ ∑ van der Waals (vdW) radii + 0.5 Å. Angle criteria: α_A···X–C/H_ between 145° and 180° (A = O, N, S, and Se; X = Br, I, S, Se, and Te). HlgBs or ChBs involving O atoms from water molecules were not considered. The vdW radii values used for O, N, S, Se, and Te were taken from the study by Álvarez [68].

This resulted in the following list of structures: 376D, 1UE2, 3IBK, 6JKN, 2H1M, 7Y8P, 3LTU, 2A0P, 4KW0, 3DW6, 3IKI, 3HG8, and 3FA1. Since 7Y8P/2A0P and 3IKI/3HG8 exhibited very similar geometrical features, we selected one structure from each pair as a representative example. Consequently, structures 376D, 1UE2, 3IBK, 6JKN, 2H1M, 7Y8P, 3LTU, 4KW0, 3DW6, 3HG8, and 3FA1 were used for calculations.

### 3.2. QM Calculations on Selected Structures

The interaction energies of the HlgB and ChB complexes studied herein were computed at the RI-MP2 [69]/def2-TZVP [70] level of theory. This level of theory has obtained success to accurately represent σ-hole-based interaction energies involving both neutral and charged electron donors [71]. The calculations were performed using TURBOMOLE version 7.2 software [72]. The interaction energy values (Δ*E*_BSSE_) were calculated as single point calculations of the energetic difference between the complex and the isolated monomers following supermolecule approximation (Δ*E*_complex_ = *E*_complex_ − *E*_monomerA_ − *E*_monomerB_), with the exception of structures 4KW0 and 3FA1 (O···Te), where the values shown in Table 1 were obtained by computing the energetic difference between the “closed” and “open” conformations of the Se and Te nucleotides (see ESI for the cartesian coordinates of both models).

In the case of the HlgBs, the computational models included the halogenated nucleobase (capped with a methyl group) and the electron donor molecule (a dimethyl phosphate group in the case of the 376D and 1UE2 structures, an adenine in the case of 3IBK, and a thymine ring attached to a sugar moiety in the case of the 6JKN structure). The capping of the nucleobase was avoided in 6JKN due to the proximity to the sp^3^ O atom from the sugar ring; therefore, a H atom was used instead of the methyl group on the nucleobase.

On the other hand, the theoretical models used to compute the ChB energies consisted of (i) the nucleobase (in 3LTU and 3HG8) or (ii) the selenated sugar moiety (in the 2H1M, 7Y8P, 3DW6, and 4KW0 structures), and (iii) the electron donor species (the same molecule in the case of the 2H1M and 7Y8P structures, a guanine/adenine ring in 3LTU, 3HG8 and 3DW6, and a phosphate group in 4KW0). In the case of the 3FA1 structure, two computational models were used; the first one contained the nucleobase and a guanine ring (−9.0 kcal/mol in Table 2), and the second one contained the nucleobase attached to sugar and phosphate groups (−15.8 kcal/mol in Table 2).

Lastly, in the 3IBK, 3LTU, 3HG8, and 3FA1 structures, additional computational models were built to estimate with higher accuracy the energetic contribution of the HlgB and ChB interaction to the stability of the supramolecular complex. To achieve this, the following modifications were carried out:

- In 3IBK, the amino group from the guanine ring was replaced by a −H atom;
- In the 3LTU, 3HG8, and 3FA1 structures, the Ch–X (Ch = S, Se, and Te; X = Me and H) moiety was replaced by a –H atom.

In all four cases, the energetic contribution of the HlgB/ChB was estimated as a difference between the interaction energies gathered in Table 1 and Table 2 and the interaction energies retrieved from these additional models, resulting in the values noted into parentheses in Table 1 and Table 2 (see also ESI for their cartesian coordinates).

In the case of I and Te, pseudopotentials [73] along with the def2-TZVP basis set were used to accelerate the calculations and account for relativistic effects, which could not be neglected. The theoretical models used to compute the interaction energies from the X-ray structures are gathered in the ESI. In all cases, the H atoms were relaxed at the BP-86 [74]-D3 [75]/def2-SVP level of theory to obtain a more reliable position before evaluating the interaction energy of the system at the RI-MP2/def2-TZVP level of theory, and the rest of the atoms were kept frozen during the optimization process.

The MEP surfaces were calculated at the RI-MP2/def2-TZVP level of theory using the Gaussian-16 calculation package [76] and the results were visualized using the Gaussview 5.0 program [77]. Bader’s “atoms in molecules” theory was used to analyze and describe the interactions discussed in this work using the AIMall calculation package [78]. The RI-MP2/def2-TZVP level of theory was also used for wavefunction analysis (also using Gaussian-16 software).

The NCIplot [79] isosurfaces acknowledge the presence of both attractive and nonattractive interactions, as denoted by the sign of the second-order density Hessian eigenvalue and the isosurface color. Noncovalent contacts are identified with the peaks that emerge in the reduced density gradient (RDG) at low densities [80]. These are plotted by mapping an isosurface of s (s = |∇ρ|/ρ4/3) for a low value of RDG. The color scheme comprises a red–yellow–green–blue scale using red for repulsive (ρ^+^ cut) and blue for attractive (ρ^−^ cut) NCI interaction density. Weak repulsive and attractive interactions are identified by yellow and green surfaces, respectively. Finally, NBO (Natural Bonding Orbital) analysis was carried out at the HF/def2-TZVP level of theory.

## 4. Conclusions

In summary, we have explored the PDB and found isolated nucleic acid structures in which halogens (Br and I) and chalcogens (S, Se, and Te) formed part of either the nucleobase or the sugar moiety. A close inspection revealed the presence of electrophilic regions over the halogen and chalcogen atoms that were interacting with electron-rich species (e.g., N and O atoms), thus directing the solid-state architecture of the NAs. Computations at the RI-MP2/def2-TZVP level of theory revealed the attractive and weak/moderately strong character of these noncovalent interactions. Furthermore, QTAIM, NCIplot, and NBO methodologies were used to further inspect the HlgBs and ChBs studied herein from a charge density perspective, extension in real space, as well as the orbital contributions implied in the formation of these supramolecular complexes. We expect that the results derived from our study will be useful for those scientists working in the fields of NA or protein engineering and σ-hole interactions, as well as in the design and synthesis of novel biomaterials based on DNA.

## Figures and Tables

**Table 1:** BSSE-corrected interaction energies of the HlgBs present in the 376D, 3IBK, 6JKN, and 1UE2 structures (ΔE_BSSE_, in kcal/mol), distance (d, in Å), and angle values (α, in °) at the RI-MP2/def2TZVP level of theory. In the case of 3IBK, the HlgB contribution to the total interaction energy is indicated in parentheses.

PDBID	ΔE_BSSE_	d	α ^1^
376D (O···Br)	−12.8	3.093	162.2
3IBK (N···Br)	−6.0 (−2.2)	2.996	157.2
6JKN (π···Br)	−2.2	3.674	174.4
1UE2 (O···I)	−16.6	3.144	168.3

^1^ Measured as the A···Hlg–C angle (A = O, N, and C; Hlg = Br and I).

**Table 2:** BSSE-corrected interaction energies of the ChBs present in the 2H1M, 7Y8P, 3LTU, 4KW0, 3DW6, 3HG8, and 3FA1 structures (ΔE_BSSE_, in kcal/mol), distance (d, in Å), and angle values (α, in °) at the RI-MP2/def2-TZVP level of theory. In the case of 3LTU, 3HG8, and 3FA1, the ChB contribution to the total interaction energy is indicated in parentheses.

PDBID	ΔE_BSSE_	d	α ^1^
2H1M (Se···Se)	−3.7	3.958	170.4
7Y8P (O···Se)	−3.1	3.407	148.4
3LTU (π···Se)	−7.8 (−2.0)	3.467	168.4
4KW0 (O···Se)	−4.3	3.089	172.6
3DW6 (N···Se)	−3.0	3.736	166.8
3HG8 (π···S)	−7.6 (−1.5)	3.467	172.1
3FA1 (O···Te)	−15.8	3.528	170.8
3FA1 (π···Te)	−9.0 (−3.4)	3.675	167.6

^1^ Measured as the A···Ch–C angle (A = Se, O, N, and C; Ch = S, Se, and Te).

**Table 3:** Donor and acceptor NBOs with indication of the second-order interaction energy E^(2)^ in the structures found during the PDB search. LP, BD, and BD* represent lone pair, bonding orbital, and antibonding orbital, respectively. Energy values are in kcal/mol.

PDBID	Donor	Acceptor	E^(2)^
376D (O···Br)	LP O	BD* Br–C	1.66
3IBK (N···Br)	LP N	BD* Br–C	3.29
6JKN (π···Br)	BD C–O	BD* Br–C	0.96
1UE2 (O···I)	LP O	BD* I–C	2.81
2H1M (Se···Se)	LP Se	BD* Se–C	0.85
7Y8P (O···Se)	LP O	BD* Se–C	0.48
3LTU (π···Se)	BD C–C BD C–N	BD* Se–C BD* Se–C	0.31 0.39
4KW0 (O···Se)	LP O	BD* Se–C	1.97
3DW6 (N···Se)	LP N	BD* Se–C	0.14
3HG8 (π···S)	BD C–N	BD* S–C	0.40
3FA1 (O···Te) 3FA1 (π···Te)	LP O BD C–N	BD* Te–H BD* Te–H	1.31 0.85

## Data Availability

All data needed to reproduce the results are gathered in the supporting information.

## Supplementary Materials

The following supporting information can be downloaded at: https://www.mdpi.com/article/10.3390/ijms241713035/s1, Cartesian coordinates of the PDB models used in Figures S1 and S2.
