# Supplementary material for: On the Importance of Halogen and Chalcogen Bonds in the Solid State of Nucleic Acids: A Combined Crystallographic and Theoretical Perspective
Source: Int J Mol Sci. 2023 Aug 22;24(17):13035. doi: 10.3390/ijms241713035 (PMC10488009; doi:10.3390/ijms241713035)
Supplement: Supplementary file 1 [file ijms-24-13035-s001.zip › ijms-2558347-supplementary.pdf]

On the Importance of Halogen and Chalcogen Bonds in the Solid State of Nucleic Acids: A Combined Crystallographic and Theoretical Perspective

*María de las Nieves Piña and Antonio Bauzá **

Department of Chemistry, Universitat de les Illes Balears, Crta. de Valldemossa km 7.5, 07122 Palma (Baleares), Spain

* E-mail: antonio.bauza@uib.es; Fax: +34-971-173426

Electronic Supporting Information

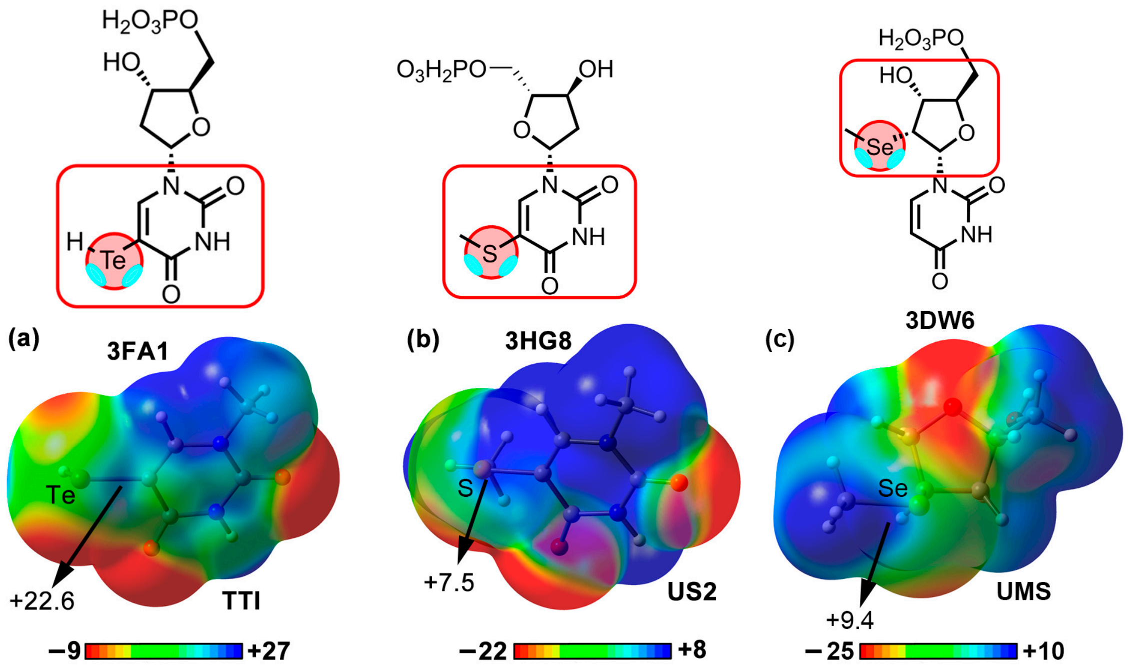

Figure S1. Molecular Electrostatic Potential (MEP) surfaces of the Se moieties present in (**a**) TTI, (**b**) US2 and (**c**) UMS ligands. Energy values at concrete regions of the surface are given in kcal/mol (0.001 a.u.). The MEP minima and maxima have been adjusted for a clear visualization of the Se σ-hole. The corresponding PDB codes are also included below the schematics of each molecule.

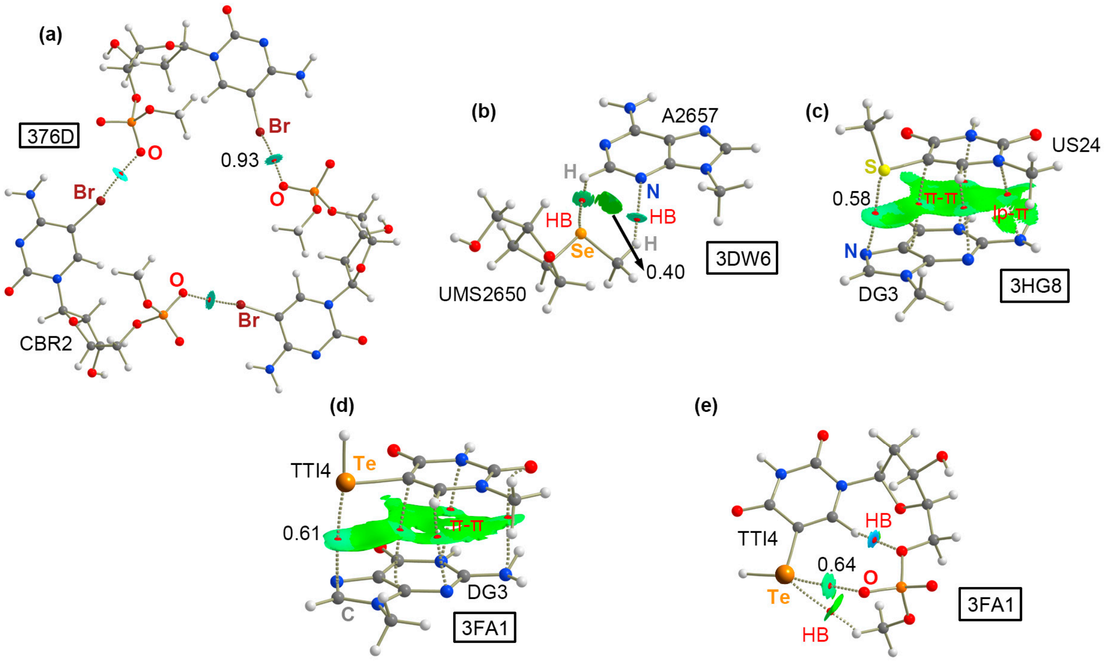

Figure S2. NCIplot analysis and AIM distribution of intermolecular bond critical points (BCP in red spheres) and bond paths in (**a**) 376D, (**b**) 3DW6, (**c**) 3HG8 and (**d, e**) 3FA1 structures. Additional interactions are denoted in red. The value of density at the BCPs characterizing the HlgB and ChB interaction is also indicated. NCIplot surfaces involving only intermolecular contacts. NCIplot color range −0.02 au ≤ (signλ_2)ρ ≤ +0.02 au. Isosurface value |RGD| = 0.5 and ρ cutoff 0.04 au.

Cartesian coordinates of the PDB models used

376D (O···Br)

C	4.0081253	2.7385083	-2.9096030
O	3.8881255	1.3865115	-2.4826038
P	3.2111271	0.7895130	-1.1596068
O	2.8081279	-0.5904840	-1.5806058
O	2.0451296	1.5795110	-0.6866079
O	4.4101245	0.7395130	-0.1086092
C	5.5781218	-0.0314853	-0.5136082
Br	-0.4928644	0.4845136	0.7013890
N	-4.4478555	-0.4664845	1.3763874
C	-3.2588585	0.1675141	1.1493881
C	-4.4938555	-1.8544813	1.5263873
O	-5.5768531	-2.4184798	1.7173866
N	-3.3248582	-2.5564797	1.4283874
C	-2.1488609	-1.8914813	1.1893880
N	-1.0728633	-2.6934794	1.1493881
C	-2.0788610	-0.4734845	1.0443882
C	-5.7588529	0.1885141	1.4943873
H	3.1688797	3.3541366	-2.5081114
H	5.2345719	-0.9801482	-0.9830729
H	6.1939092	0.5322006	-1.2509367
H	-3.3126651	1.2629263	1.0474767
H	-1.2528637	-3.6963299	1.1658347
H	-6.2226930	-0.0820390	2.4622273
H	4.9689497	3.2049015	-2.5758897

H	3.9792390	2.7669743	-4.0213822
H	6.1826885	-0.2360793	0.3950121
H	-0.1692667	-2.3381871	0.8259676
H	-5.6298425	1.2857032	1.4197109
H	-6.4337419	-0.1727871	0.6938938

3IBK (N···Br)

C	-3.0992510	1.3090971	4.8279601
N	-2.2962516	1.3120971	3.5899608
C	-2.1332516	0.2750977	2.6969613
N	-1.2862520	0.5400975	1.7219619
C	-0.8712524	1.8440967	2.0029617
C	0.0297472	2.7080963	1.3599621
N	0.6807468	2.3850965	0.2639627
N	0.2177470	3.9220955	1.8799618
C	-0.4442526	4.2510954	2.9899612
N	-1.3062520	3.5260958	3.6989608
C	-1.4782520	2.3210965	3.1449611
C	1.1557466	-4.3778997	-4.3820347
N	1.4857464	-3.1319005	-3.6480351
C	2.4897458	-2.3039009	-4.1190349
O	3.1967454	-2.5779007	-5.0740343
N	2.6317458	-1.1309016	-3.4330352
C	1.8777462	-0.7029019	-2.3440358
O	2.1237460	0.3830975	-1.8140361
C	0.8437468	-1.6089013	-1.9220361
C	0.6777468	-2.7589007	-2.5640357
Br	-0.2242526	-1.1589015	-0.5060369
H	-2.5482794	1.8697100	5.6063691
H	-2.6730469	-0.6737462	2.8263705
H	0.6697647	1.4464654	-0.1437485
H	-0.2437456	5.2697364	3.3742108
H	-0.0977474	-3.4856838	-2.2852579
H	1.3550622	3.0535010	-0.1079290
H	-4.0787798	1.8026268	4.6659181
H	-3.2588304	0.2656431	5.1563385
H	0.8170298	-4.1389925	-5.4096296
H	2.0549930	-5.0185083	-4.4582617
H	0.3583153	-4.9093729	-3.8359674
H	3.3746482	-0.5037066	-3.7547505

3IBK (–H replacing –NH_2 group)

C	-3.09925100	1.30909710	4.82796010
N	-2.29625160	1.31209710	3.58996080
C	-2.13325160	0.27509770	2.69696130
N	-1.28625200	0.54009750	1.72196190
C	-0.87125240	1.84409670	2.00296170
C	0.02974720	2.70809630	1.35996210
N	0.21774700	3.92209550	1.87996180
C	-0.44425260	4.25109540	2.98996120
N	-1.30625200	3.52609580	3.69896080
C	-1.47825200	2.32109650	3.14496110
C	1.15574660	-4.37789970	-4.38203470
N	1.48574640	-3.13190050	-3.64803510
C	2.48974580	-2.30390090	-4.11903490
O	3.19674540	-2.57790070	-5.07403430
N	2.63174580	-1.13090160	-3.43303520
C	1.87774620	-0.70290190	-2.34403580
O	2.12374600	0.38309750	-1.81403610
C	0.84374680	-1.60890130	-1.92203610
C	0.67774680	-2.75890070	-2.56403570

Br	-0.22425260	-1.15890150	-0.50603690
H	-2.54827940	1.86971000	5.60636910
H	-2.67304690	-0.67374620	2.82637050
H	-0.24374560	5.26973640	3.37421080
H	-0.09774740	-3.48568380	-2.28525790
H	-4.07877980	1.80262680	4.66591810
H	-3.25883040	0.26564310	5.15633850
H	0.81702980	-4.13899250	-5.40962960
H	2.05499300	-5.01850830	-4.45826170
H	0.35831530	-4.90937290	-3.83596740
H	3.37464820	-0.50370660	-3.75475050
H	0.49587657	2.47682167	0.57520353

6JKN (π···Br)

N	2.0103577	0.7893834	2.3340262
C	0.9733607	0.3173846	3.0100242
N	1.0943604	0.2023852	4.3300206
C	2.3363567	0.6373838	4.5450198
C	2.9423550	1.0133828	3.2570236
N	4.1973516	1.4883815	3.2210237
C	4.8953497	1.6293811	4.3590204
N	6.1503460	2.1093796	4.2860207
N	4.3863509	1.2873821	5.5620171
C	3.1583545	0.7943834	5.7400167
O	2.6843557	0.4873844	6.8750132
Br	-0.7126345	-0.2426136	2.1470267
C	-0.4016355	1.2603823	-4.2779553
C	0.3153625	0.0913854	-3.6049573
O	-0.1576363	-0.0376144	-2.2279612
C	0.0663630	-1.2726106	-4.2579553
O	1.2053598	-2.1056084	-4.1119558
C	-1.0916335	-1.8226093	-3.4509578
C	-0.7486345	-1.3156106	-2.0519616
N	-1.9466313	-1.1996109	-1.1849640
C	-2.1546305	-2.1626084	-0.2219668
O	-1.3816328	-3.0936056	-0.0309673
N	-3.2946273	-1.9986086	0.5220312
C	-4.2436246	-1.0026115	0.3890317
O	-5.2506220	-0.9516116	1.0980297
C	-3.9846255	-0.0386144	-0.6579654
C	-4.9486226	1.0813826	-0.8949648
C	-2.8636285	-0.1826136	-1.3979633
H	6.8289447	1.9111044	5.0202751
H	-0.3207603	2.1701199	-3.6521701
H	0.0389412	1.4788959	-5.2724164
H	1.4101011	0.2895768	-3.5613032
H	-0.2090566	-1.1494168	-5.3345497
H	-1.1666258	-2.9244658	-3.5050369
H	-2.0455652	-1.3777400	-3.7977965
H	-0.0704051	-2.0127412	-1.5188658
H	-5.9655856	0.6895521	-1.1048729
H	-5.0492900	1.7123552	0.0129265
H	-4.6294568	1.7223089	-1.7390275
H	-2.5721905	0.5275104	-2.1854177
H	-1.4791147	1.0361552	-4.4192004
H	1.9419231	-1.6869102	-4.5923412
H	4.9131527	1.4191990	6.4335862
H	6.5162230	2.2119547	3.3394480
H	-3.4503189	-2.7004257	1.2519342
H	2.0667007	0.9247418	1.3227400

6JKN (π···Br) Trimer

```
C    -22.89100000   30.64500000   59.81900000
N    -22.52600000   29.25300000   60.19300000
C    -21.89200000   29.00600000   61.39100000
O    -21.71000000   29.86400000   62.24000000
N    -21.52000000   27.69400000   61.58100000
C    -21.68200000   26.64300000   60.68200000
O    -21.30600000   25.50400000   60.91700000
C    -22.33800000   26.98000000   59.46200000
C    -22.57600000   25.92300000   58.43200000
C    -22.70100000   28.25200000   59.26400000
C    -14.02400000   30.10500000   57.82900000
N   -14.28900000   30.14900000   56.38100000
C    -15.21600000   29.48700000   55.70500000
N    -15.25500000   29.64900000   54.38500000
C    -14.25700000   30.50700000   54.17000000
C    -13.62900000   30.84400000   55.45800000
N    -12.59000000   31.69300000   55.49400000
C    -12.11900000   32.22700000   54.35600000
N    -11.07500000   33.07400000   54.42900000
N    -12.66900000   31.95700000   53.15300000
C    -13.71000000   31.14000000   52.97500000
O    -14.21300000   30.88300000   51.84000000
Br   -16.54400000   28.30600000   56.56800000
C    -17.49100000   28.81200000   60.76700000
N    -17.99000000   27.71700000   59.90000000
C    -18.92800000   28.01800000   58.93700000
O    -19.34800000   29.15300000   58.74600000
N    -19.35600000   26.94900000   58.19300000
C    -18.96800000   25.62900000   58.32600000
O    -19.42700000   24.73100000   57.61700000
C    -18.00400000   25.37100000   59.37300000
C    -17.51600000   23.97600000   59.61000000
C    -17.56800000   26.41400000   60.11300000
H    -22.76264060   31.27489450   60.71591460
H    -21.65020130   25.37336550   58.16548340
H    -23.27614130   25.15250620   58.82127540
H    -23.01827720   26.35023980   57.51078800
H    -23.19236200   28.57655860   58.33370540
H    -14.91564800   30.45138930   58.38524170
H    -10.80246020   33.34222340   55.37433660
H    -17.60791410   29.76161290   60.21597170
H    -16.76224400   23.93847080   60.41962240
H    -18.36439760   23.31297000   59.87903300
H    -17.07418410   23.55189540   58.68473760
H    -16.84168200   26.28932870   60.93041390
H    -13.78345810   29.06914660   58.13381650
H    -13.16562520   30.77184470   58.02807250
H    -16.42617580   28.63172000   61.00469820
H    -18.08359760   28.87165250   61.70278360
H    -22.22046200   31.00194120   59.01234040
H    -23.94575250   30.67153930   59.48385080
H    -10.89697090   33.75263370   53.68979410
H    -12.29108170   32.34834690   52.28208660
H    -20.05827510   27.16474200   57.48026970
H    -21.07703390   27.48281190   62.47875570
```

1UE2 (O···I)

```
C    -3.1750821   -4.7457083    1.6070991
N    -1.8920867   -4.0537107    1.4430997
```

C	-0.7210910	-4.8217080	1.4850995
O	-0.8270906	-6.0587034	1.6420989
N	0.4809044	-4.2197101	1.3511000
C	0.5539043	-2.8997150	1.1531007
N	1.7578999	-2.3577170	1.0501012
C	-0.6300916	-2.0827179	1.0711011
C	-1.8210871	-2.6967158	1.2391004
I	-0.5420918	-0.0467254	0.5781028
C	-0.2910927	4.6832574	-2.9708842
O	0.9299030	4.0612595	-2.5908855
P	1.0119027	3.1752628	-1.2508905
O	-0.3480923	2.7912642	-0.7598922
O	2.0108990	2.1232667	-1.5198894
O	1.6369004	4.1782591	-0.1918943
C	2.8878957	4.8032569	-0.4628933
H	-3.1510426	-5.3600498	2.5284381
H	2.5617666	-2.9845370	1.0430729
H	-2.7765634	-2.1532155	1.1930098
H	-1.1572851	4.1400059	-2.5330729
H	3.6327138	4.0443017	-0.7975792
H	2.7972871	5.5737681	-1.2654088
H	-0.3679850	4.6802377	-4.0815533
H	-0.3399091	5.7468781	-2.6282998
H	3.2460473	5.2938198	0.4678642
H	1.8710845	-1.3765208	0.7799853
H	-3.3494943	-5.4383053	0.7587168
H	-3.9899607	-3.9987827	1.6610344

2H1M (Se···Se)

C	3.46994320	-3.12516580	-1.13296240
O	4.44394320	-2.10716580	-0.88196240
C	2.56194320	-3.18216580	0.07903760
Se	0.98094320	-2.06416580	-0.24696240
C	-0.09005680	-3.35916580	-1.27796240
C	3.40194320	-2.55916580	1.16303760
C	4.27094320	-1.57416580	0.43103760
C	5.58294320	-1.47616580	1.17103760
O	6.40094320	-0.59216580	0.43803760
C	0.97194320	4.56783420	-1.13296240
O	-0.39705680	4.90183420	-0.88196240
C	1.47494320	3.80983420	0.07903760
Se	1.29694320	1.88083420	-0.24696240
C	2.95394320	1.60083420	-1.27796240
C	0.51494320	4.22583420	1.16303760
C	-0.77205680	4.48583420	0.43103760
C	-1.51305680	5.57283420	1.17103760
O	-2.68805680	5.83883420	0.43803760
H	2.92627660	-2.90415630	-2.07657710
H	2.20802250	-4.20399320	0.31917360
H	0.43208860	-3.65980780	-2.20795070
H	-0.32599870	-4.24926750	-0.66324260
H	-1.03345730	-2.84085220	-1.53918460
H	4.03640470	-3.34867600	1.62500110
H	3.80759010	-0.56078140	0.37416470
H	5.37479100	-1.12649390	2.21552110
H	6.02506460	-2.50254090	1.23684210
H	1.05277260	3.98693280	-2.07688480
H	2.53691910	4.01392170	0.31936260
H	2.95797370	2.21273910	-2.20155080
H	3.84323640	1.83011840	-0.65954670
H	2.96965960	0.52704450	-1.54986600

H	0.88160780	5.17035130	1.62393500
H	-1.41741730	3.57762290	0.37445770
H	-1.71131570	5.21788530	2.21564930
H	-0.84585990	6.46934650	1.23640640
H	-3.16155470	6.57338990	0.86711820
H	7.27141640	-0.54375660	0.87157400
H	2.80394090	-2.10711230	1.97975540
H	0.42215400	3.48187610	1.97941140
H	4.00154750	-4.09952560	-1.27874050
H	1.54963530	5.51579260	-1.27761640

7Y8P (O···Se)

C	1.7500000	2.2470000	26.8570000
C	2.7190000	2.5480000	28.0100000
C	3.4340000	1.2520000	28.5080000
C	4.0560000	0.5980000	27.2760000
O	3.6740000	3.4870000	27.5510000
O	4.4090000	1.6830000	29.4540000
Se	2.7650000	0.9030000	25.8910000
C	3.3490000	5.5960000	31.1720000
C	2.4470000	5.5020000	32.3830000
O	3.0900000	5.8180000	33.6070000
C	1.2580000	6.4320000	32.0150000
O	1.7940000	7.7930000	31.9630000
C	0.5760000	6.1580000	30.7160000
Se	2.1470000	5.6870000	29.6580000
H	1.5960248	3.1484475	26.2357390
H	2.1455912	2.9804211	28.8739048
H	2.6825455	0.5656738	28.9660170
H	5.0121597	1.1059698	27.0364026
H	4.4138592	3.4173704	28.1924783
H	3.9573830	6.5203875	31.2254615
H	2.0390836	4.4581582	32.4251053
H	0.5072616	6.3760488	32.8524848
H	2.3863412	7.8512616	32.7413728
H	0.0930767	7.0756092	30.3337464
H	2.4726761	5.5866035	34.3255638
H	4.2389428	-0.4853903	27.4075076
H	-0.1446627	5.3203862	30.7711112
H	0.7820454	1.8469513	27.2151575
H	3.9320412	1.9076678	30.2741223
H	4.0217894	4.7238895	31.0541111

3LTU (π···Se)

C	41.1170000	29.0730000	20.5660000
N	40.5160000	29.8420000	21.6510000
C	40.9340000	31.0490000	22.1620000
N	40.1780000	31.4950000	23.1260000
C	39.1960000	30.5200000	23.2640000
C	38.0920000	30.4440000	24.1500000
O	37.7590000	31.2540000	25.0220000
N	37.3370000	29.2850000	23.9620000
C	37.6110000	28.3150000	23.0240000
N	36.7810000	27.2650000	22.9760000
N	38.6420000	28.3700000	22.1880000
C	39.3890000	29.4940000	22.3640000
N	35.5570000	29.2340000	20.3800000
C	36.5520000	30.1750000	20.1790000
C	34.5900000	29.4110000	21.3500000
O	33.6890000	28.6230000	21.5600000
N	34.7120000	30.5620000	22.0860000

C	35.6750000	31.5390000	21.9320000
O	35.6830000	32.5310000	22.6460000
C	36.6570000	31.3000000	20.8990000
Se	38.0580000	32.5630000	20.5790000
C	37.3010000	33.8890000	19.4150000
C	35.5100000	27.9980000	19.5340000
H	40.7215199	28.0412875	20.6114003
H	41.8232379	31.5535711	21.7579276
H	35.8033212	27.3697237	23.2536546
H	37.2612302	29.9319124	19.3736026
H	36.9767521	33.4784560	18.4383834
H	36.4600866	34.4215949	19.9005934
H	38.1268665	34.6085486	19.2482122
H	34.6694540	27.3830770	19.8989729
H	35.3433695	28.2716997	18.4739408
H	36.4687615	27.4523984	19.6353694
H	42.2182694	29.0484711	20.6770034
H	40.8607586	29.5124519	19.5794726
H	36.9420074	26.6218375	22.2005675
H	36.5507293	29.1894458	24.6107216
H	33.9961581	30.7244645	22.7999706

3LTU (-H replacing a -SeCH$_3$ group)

C	41.11700000	29.07300000	20.56600000
N	40.51600000	29.84200000	21.65100000
C	40.93400000	31.04900000	22.16200000
N	40.17800000	31.49500000	23.12600000
C	39.19600000	30.52000000	23.26400000
C	38.09200000	30.44400000	24.15000000
O	37.75900000	31.25400000	25.02200000
N	37.33700000	29.28500000	23.96200000
C	37.61100000	28.31500000	23.02400000
N	36.78100000	27.26500000	22.97600000
N	38.64200000	28.37000000	22.18800000
C	39.38900000	29.49400000	22.36400000
N	35.55700000	29.23400000	20.38000000
C	36.55200000	30.17500000	20.17900000
C	34.59000000	29.41100000	21.35000000
O	33.68900000	28.62300000	21.56000000
N	34.71200000	30.56200000	22.08600000
C	35.67500000	31.53900000	21.93200000
O	35.68300000	32.53100000	22.64600000
C	36.65700000	31.30000000	20.89900000
C	35.51000000	27.99800000	19.53400000
H	40.72151990	28.04128750	20.61140030
H	41.82323790	31.55357110	21.75792760
H	35.80332120	27.36972370	23.25365460
H	37.26123020	29.93191240	19.37360260
H	34.66945400	27.38307700	19.89897290
H	35.34336950	28.27169970	18.47394080
H	36.46876150	27.45239840	19.63536940
H	42.21826940	29.04847110	20.67700340
H	40.86075860	29.51245190	19.57947260
H	36.94200740	26.62183750	22.20056750
H	36.55072930	29.18944580	24.61072160
H	33.99615810	30.72446450	22.79997060
H	37.44053684	32.00635762	20.72003370

4KW0 (O···Se) (closed conformation)

C	8.85700000	25.03400000	0.86000000
O	9.64300000	26.18600000	0.61200000

C	11.45000000	23.56100000	-3.54000000
O	11.71400000	24.31100000	-2.38200000
C	10.61600000	24.49300000	-4.41900000
Se	8.74400000	24.80000000	-3.85500000
C	7.63900000	23.68200000	-5.06300000
C	11.44400000	25.76000000	-4.23000000
O	12.50000000	25.71000000	-5.14800000
C	12.03700000	25.64500000	-2.82600000
C	11.52300000	26.63700000	-1.79900000
O	10.11800000	26.63500000	-1.81000000
P	9.32300000	27.22600000	-0.56100000
O	7.87900000	27.15300000	-0.88100000
O	9.95200000	28.50200000	-0.14800000
H	7.77660540	25.26952620	0.76798950
H	12.39018100	23.29672520	-4.08904570
H	10.59643730	24.19886030	-5.48698470
H	6.60065340	23.92766890	-4.76792020
H	7.81253770	22.59836870	-4.91355270
H	7.78626980	23.94873830	-6.12839360
H	10.83309170	26.68732920	-4.33925520
H	13.14240250	25.77183010	-2.92095420
H	11.91292140	26.33079100	-0.80204990
H	11.92239100	27.65048240	-2.03491900
H	12.13628150	25.88087280	-6.03537720
H	9.10649650	24.22947150	0.13469890
H	9.08876940	24.68746210	1.88602400
H	10.93327640	22.62642740	-3.24339780

4KW0 (O···Se) (open conformation)

C	14.43056971	29.05444582	-0.23393128
O	13.02607098	28.88280894	-0.29971956
C	11.45000000	23.56100000	-3.54000000
O	11.71400000	24.31100000	-2.38200000
C	10.61600000	24.49300000	-4.41900000
Se	8.74400000	24.80000000	-3.85500000
C	7.63900000	23.68200000	-5.06300000
C	11.44400000	25.76000000	-4.23000000
O	12.50000000	25.71000000	-5.14800000
C	12.03700000	25.64500000	-2.82600000
C	11.52300000	26.63700000	-1.79900000
O	12.26877990	26.51203919	-0.61479261
P	12.25462503	27.69840327	0.44993595
O	13.06515879	27.26158786	1.60971710
O	10.86263327	28.17408392	0.62450073
H	14.79906487	28.87750547	0.79761825
H	12.39018100	23.29672520	-4.08904570
H	10.59643730	24.19886030	-5.48698470
H	6.60065340	23.92766890	-4.76792020
H	7.81253770	22.59836870	-4.91355270
H	7.78626980	23.94873830	-6.12839360
H	10.83309170	26.68732920	-4.33925520
H	13.14240250	25.77183010	-2.92095420
H	11.62717693	27.65911484	-2.22811602
H	10.44127465	26.44462567	-1.61143694
H	12.13628150	25.88087280	-6.03537720
H	14.94171328	28.34359283	-0.91876452
H	14.65420930	30.09229547	-0.54918398
H	10.93327640	22.62642740	-3.24339780

3DW6 (N···Se)

O	-1.7036064	-3.7728208	5.1070992

C	-2.0536060	-3.6638209	3.7551008
C	-1.5166067	-2.3448224	3.1821014
O	-2.0806060	-1.2358236	3.9421005
C	-0.0116083	-2.1868225	3.3761012
C	0.1473914	-0.7058242	3.1761014
Se	0.0183917	-0.1928248	1.3971034
C	-1.0416071	-0.2178248	4.0141004
C	0.3553913	1.6011732	1.3871034
C	-1.0336072	3.3361712	-2.6778920
N	-0.0966083	2.6791720	-3.5818910
C	0.2233914	3.0151716	-4.8688896
N	1.0923905	2.1991726	-5.4208889
C	1.3533901	1.2701736	-4.4258901
C	2.1833892	0.1351748	-4.3688901
N	2.9463884	-0.2728246	-5.3858889
N	2.2033892	-0.5678244	-3.2168914
C	1.4373901	-0.1698248	-2.1968925
N	0.6173909	0.8741740	-2.1328926
C	0.6253909	1.5521732	-3.2888913
H	-3.1630422	-3.6745049	3.6070433
H	-1.6347501	-4.4894169	3.1228953
H	-1.8081215	-2.2834477	2.1063979
H	0.2440499	-2.4734927	4.4149008
H	1.1009259	-0.3252534	3.6049086
H	-1.4555046	0.7547457	3.6678430
H	1.3522073	1.8734075	1.7969746
H	-0.4126640	2.2147079	1.9064076
H	0.3501677	1.8703853	0.3103389
H	-1.6323738	2.5685806	-2.1510091
H	-0.2289312	3.8902231	-5.3575259
H	3.5209564	-1.1058917	-5.2686956
H	1.5017739	-0.7846655	-1.2792919
H	-0.7425189	-0.0940093	5.0832426
H	-2.0018310	-4.6410052	5.4277614
H	-1.7049192	3.9950095	-3.2590712
H	-0.4965970	3.9393098	-1.9175404
H	2.9514480	0.2441209	-6.2625575
H	0.5849991	-2.8096842	2.6772552

3HG8 (π···S)

C	0.90053550	-3.62303830	0.88043130
N	0.11653780	-2.98804010	-0.17556540
C	-1.10745840	-3.37703890	-0.67156390
N	-1.55145710	-2.61104120	-1.62956100
C	-0.55246000	-1.65304430	-1.78656050
C	-0.45646050	-0.54704770	-2.67055770
O	-1.26845780	-0.18604880	-3.52955510
N	0.72753610	0.17395010	-2.49055830
C	1.70153290	-0.12904890	-1.56756110
N	2.78552950	0.66594850	-1.51556140
N	1.62753320	-1.16004580	-0.73856370
C	0.47953670	-1.87204360	-0.89956320
N	0.84653560	1.92194470	1.13343060
C	-0.10365171	0.87388838	1.41422753
C	0.63353620	2.82594190	0.16143350
O	1.41653390	3.78293900	-0.03356590
N	-0.51746010	2.73294230	-0.57756430
C	-1.48645730	1.77494510	-0.38356480
O	-2.49545400	1.78594500	-1.11856250
C	-1.30145780	0.82194800	0.62643200
S	-2.39545430	-0.44604800	0.97943090

C	-3.59545070	0.38494940	1.96042800
C	2.09253170	1.91494470	1.91442800
H	1.97307220	-3.42136290	0.69883840
H	-1.62842810	-4.25335990	-0.25962580
H	3.43183410	0.46323560	-0.75300450
H	0.10598989	0.12576038	2.23255213
H	-4.37339420	-0.36515770	2.20771490
H	-3.18237750	0.78467420	2.91014420
H	-4.08176850	1.20438740	1.39235580
H	2.71007230	2.73471500	1.50833490
H	1.87027830	2.09721040	2.98368750
H	2.58832820	0.93149820	1.79515880
H	0.62537670	-3.22014520	1.87762320
H	0.72465010	-4.71539760	0.87101270
H	2.71611030	1.65061340	-1.77936190
H	0.83610030	0.96199040	-3.13514800
H	-0.68302240	3.45085250	-1.28548580

3HG8 (-H replacing a -SCH_3 group)

C	0.90053550	-3.62303830	0.88043130
N	0.11653780	-2.98804010	-0.17556540
C	-1.10745840	-3.37703890	-0.67156390
N	-1.55145710	-2.61104120	-1.62956100
C	-0.55246000	-1.65304430	-1.78656050
C	-0.45646050	-0.54704770	-2.67055770
O	-1.26845780	-0.18604880	-3.52955510
N	0.72753610	0.17395010	-2.49055830
C	1.70153290	-0.12904890	-1.56756110
N	2.78552950	0.66594850	-1.51556140
N	1.62753320	-1.16004580	-0.73856370
C	0.47953670	-1.87204360	-0.89956320
N	0.84653560	1.92194470	1.13343060
C	-0.10365171	0.87388838	1.41422753
C	0.63353620	2.82594190	0.16143350
O	1.41653390	3.78293900	-0.03356590
N	-0.51746010	2.73294230	-0.57756430
C	-1.48645730	1.77494510	-0.38356480
O	-2.49545400	1.78594500	-1.11856250
C	-1.30145780	0.82194800	0.62643200
C	2.09253170	1.91494470	1.91442800
H	1.97307220	-3.42136290	0.69883840
H	-1.62842810	-4.25335990	-0.25962580
H	3.43183410	0.46323560	-0.75300450
H	0.10598989	0.12576038	2.23255213
H	2.71007230	2.73471500	1.50833490
H	1.87027830	2.09721040	2.98368750
H	2.58832820	0.93149820	1.79515880
H	0.62537670	-3.22014520	1.87762320
H	0.72465010	-4.71539760	0.87101270
H	2.71611030	1.65061340	-1.77936190
H	0.83610030	0.96199040	-3.13514800
H	-0.68302240	3.45085250	-1.28548580
H	-1.98540324	0.02922144	0.84712008

3FA1 (O···Te)

C	35.44200000	23.68400000	3.30300000
N	34.65800000	24.18600000	2.18000000
C	33.46200000	23.70800000	1.70000000
N	33.00100000	24.40300000	0.69500000
C	33.95400000	25.39500000	0.49900000
C	34.00200000	26.45800000	-0.44300000

O	33.18700000	26.74200000	-1.32900000
N	35.14800000	27.23300000	-0.28200000
C	36.12500000	27.01300000	0.65600000
N	37.15900000	27.86300000	0.64700000
N	36.09100000	26.03000000	1.53700000
C	34.98500000	25.26800000	1.40300000
N	35.17900000	28.96100000	3.59200000
C	35.07100000	29.89400000	2.58500000
O	35.90700000	30.76200000	2.38100000
N	33.93500000	29.78100000	1.82400000
C	32.92000000	28.85500000	1.96600000
O	31.95300000	28.88500000	1.20300000
C	33.09600000	27.91000000	3.04500000
C	34.20300000	28.00700000	3.79400000
C	36.37600000	29.00000000	4.44800000
Te	31.59400000	26.42300000	3.37100000
H	36.41091270	24.21661470	3.30784070
H	32.97398020	22.83697830	2.15874200
H	37.80462140	27.76063240	1.43095290
H	34.42304210	27.30776330	4.61463040
H	36.98617050	29.86211880	4.12887150
H	36.08591650	29.12230710	5.51028830
H	36.95422950	28.06105370	4.32915830
H	34.91806470	23.87302560	4.26276820
H	35.62405170	22.59628800	3.19827960
H	37.01985440	28.82864660	0.34349710
H	35.23827040	27.98763820	-0.96824950
H	33.81272040	30.47814300	1.08382000
H	30.77206314	27.45084775	4.42934585

3FA1 (-H replacing a SeH group)

C	35.44200000	23.68400000	3.30300000
N	34.65800000	24.18600000	2.18000000
C	33.46200000	23.70800000	1.70000000
N	33.00100000	24.40300000	0.69500000
C	33.95400000	25.39500000	0.49900000
C	34.00200000	26.45800000	-0.44300000
O	33.18700000	26.74200000	-1.32900000
N	35.14800000	27.23300000	-0.28200000
C	36.12500000	27.01300000	0.65600000
N	37.15900000	27.86300000	0.64700000
N	36.09100000	26.03000000	1.53700000
C	34.98500000	25.26800000	1.40300000
N	35.17900000	28.96100000	3.59200000
C	35.07100000	29.89400000	2.58500000
O	35.90700000	30.76200000	2.38100000
N	33.93500000	29.78100000	1.82400000
C	32.92000000	28.85500000	1.96600000
O	31.95300000	28.88500000	1.20300000
C	33.09600000	27.91000000	3.04500000
C	34.20300000	28.00700000	3.79400000
C	36.37600000	29.00000000	4.44800000
H	36.41091270	24.21661470	3.30784070
H	32.97398020	22.83697830	2.15874200
H	37.80462140	27.76063240	1.43095290
H	34.42304210	27.30776330	4.61463040
H	36.98617050	29.86211880	4.12887150
H	36.08591650	29.12230710	5.51028830
H	36.95422950	28.06105370	4.32915830
H	34.91806470	23.87302560	4.26276820
H	35.62405170	22.59628800	3.19827960

H	37.01985440	28.82864660	0.34349710
H	35.23827040	27.98763820	-0.96824950
H	33.81272040	30.47814300	1.08382000
H	32.34449515	27.16600019	3.20810958

3FA1 (closed conformation)

C	34.1870000	23.7140000	5.3130000
O	34.2610000	23.9860000	6.7170000
N	35.1790000	28.9610000	3.5920000
C	35.0710000	29.8940000	2.5850000
O	35.9070000	30.7620000	2.3810000
N	33.9350000	29.7810000	1.8240000
C	32.9200000	28.8550000	1.9660000
O	31.9530000	28.8850000	1.2030000
C	33.0960000	27.9100000	3.0450000
C	34.2030000	28.0070000	3.7940000
C	36.3760000	29.0000000	4.4480000
C	36.2870000	29.9930000	5.6080000
C	35.8610000	29.1170000	6.7700000
O	36.3170000	29.6430000	8.0190000
C	36.5700000	27.8070000	6.4610000
O	36.5380000	27.7050000	5.0210000
C	35.9390000	26.5810000	7.0810000
O	34.5550000	26.5040000	6.7310000
P	33.6220000	25.3440000	7.3060000
O	32.2720000	25.5120000	6.7120000
O	33.7680000	25.3050000	8.7830000
Te	31.5940000	26.4230000	3.3710000
H	33.1353625	23.7743621	4.9549109
H	34.4036683	27.3307351	4.6425691
H	37.2186559	29.2424404	3.7669756
H	37.2937401	30.4028193	5.8316201
H	35.6131463	30.8407794	5.3857035
H	34.7617394	28.9404084	6.7664826
H	35.8486193	29.1418603	8.7127899
H	37.6252384	27.8935898	6.8180674
H	36.0387488	26.6275056	8.1926002
H	36.4764529	25.6638540	6.7463450
H	34.5877779	22.6929008	5.1371701
H	34.7904966	24.4389225	4.7144550
H	33.8294879	30.4678231	1.0729630
H	31.0510918	26.6989437	1.7938299

3FA1 (open conformation)

C	35.18803278	25.94751660	10.74839199
O	35.10662083	25.03351712	9.64900441
N	35.17900000	28.96100000	3.59200000
C	35.07100000	29.89400000	2.58500000
O	35.90700000	30.76200000	2.38100000
N	33.93500000	29.78100000	1.82400000
C	32.92000000	28.85500000	1.96600000
O	31.95300000	28.88500000	1.20300000
C	33.09600000	27.91000000	3.04500000
C	34.20300000	28.00700000	3.79400000
C	36.37600000	29.00000000	4.44800000
C	36.28700000	29.99300000	5.60800000
C	35.86100000	29.11700000	6.77000000
O	36.31700000	29.64300000	8.01900000
C	36.57000000	27.80700000	6.46100000
O	36.53800000	27.70500000	5.02100000
C	35.93900000	26.58100000	7.08100000

O	36.81772350	26.00743748	8.05195626
P	36.43558428	24.65964623	8.81624832
O	37.51927546	24.36662813	9.78749177
O	36.06143397	23.63306516	7.81100762
Te	31.59400000	26.42300000	3.37100000
H	35.96115416	25.61862903	11.47772670
H	34.40366830	27.33073510	4.64256910
H	37.21865590	29.24244040	3.76697560
H	37.29374010	30.40281930	5.83162010
H	35.61314630	30.84077940	5.38570350
H	34.76173940	28.94040840	6.76648260
H	35.84861930	29.14186030	8.71278990
H	37.62523840	27.89358980	6.81806740
H	35.72106606	25.82693953	6.28622632
H	34.96357725	26.84842351	7.54901869
H	34.19367083	25.98326778	11.24250616
H	35.45474083	26.97979214	10.41484901
H	33.82948790	30.46782310	1.07296300
H	31.05109180	26.69894370	1.79382990
